# The Prevalence and Predictors of Iron Deficiency Anemia among Rural Infants in Nablus Governorate

**Published:** 2018-06-13

**Authors:** Rania Wasef Salah, Ali Abdel Halim Hasab, Nessrin Ahmed El-Nimr, Dalia Ibrahim Tayel

**Affiliations:** ^1^ Public Health Department, Faculty of Medicine and Health Sciences, An-Najah National University; ^2^ Department of Epidemiology, High Institute of Public Health, Alexandria University, Egypt; ^3^ Department of Nutrition, High Institute of Public Health, Alexandria University, Egypt

**Keywords:** Infants, Iron deficiency anemia, Mean corpuscular volume, Prevalence, Risk factors

## Abstract

**Background:** Iron deficiency anemia (IDA) in infants and young children remains a significant public health problem in most developing countries. IDA had short and long-term adverse impacts on infants’ health and development. We aimed to assess the frequency of IDA and associated risk factors among infants aged between 9-12 months in rural areas of Nablus Governorate.

**Study design:** A cross-sectional study.

**Methods:** The study was conducted between Jan and Mar 2015. A random sample of 654 infants aged 9-12 months were selected from thirty villages in Nablus Governorate, Central Highlands of the West Bank, north of Jerusalem. Data were collected using pre-designed structured interviewing questionnaire, complete blood count analysis and anthropometric measurements were done.

**Results:** The prevalence of anemia and IDA among infants was 34.6%, and 32.6%, respectively. Predictors of IDA were increased in infants’ age OR=1.19 (95% CI: 1.02, 1.40), maternal anemia during the third trimester OR=2.39 (95% CI: 1.55, 3.71), birth spacing less than three years OR=2.86 (95%CI: 1.58, 5.18), exclusive breastfeeding during the first six months OR=2.40 (95% CI: 1.46, 3.95), early OR=1.64 (95%CI: 1.03, 2.613) and late introduction of complementary feeding OR=2.26 (95% CI: 1.27, 4.05), and non-compliance to iron supplement in the correct frequency and duration during pregnancy OR=1.81 (95% CI: 1.19, 2.75).

**Conclusions:** Different dietary and non-dietary risk factors for IDA should be considered for any intervention aimed to reduce the prevalence of IDA among infants

## Introduction

Anemia is a public health problem that affects low, middle and high-income countries^1^. Iron deficiency is the top ranking cause of anemia globally^2^, approximately half of cases of anemia are considered to be due to iron deficiency^1^. Young children had the highest prevalence of Iron deficiency anemia (IDA)^3^. The African region had the highest proportion of children affected with anemia (62.3%), and the Eastern Mediterranean Region had the next highest anemia burden for children (48.6%), while the lowest prevalence was in the Western Pacific Region (21.9%). Globally 42.6% of children less than five years had anemia^1^.

IDA in infants may occur as a consequence of decreased iron intake; infants at the age of six months should receive iron-rich complementary foods, such as meat products and iron-fortified food^4^. Prolonged breast or bottle feeding without complementary feeds beyond six months of age ^5^ and a diet rich in non-bioavailable iron will lead to IDA^6^. Furthermore, it may be due to factors such as poor absorption, increased iron loss, or increased iron requirement^7^.

IDA in infancy has long-term adverse consequences, it can lead to impairment in mental and motor functioning at five years of age ^8^, adversely affects at least one aspect of the central nervous system development that lasts at least to four years of age ^9^ and significantly impairs growth^10^. Moreover, children who were anemic in infancy continue to have poorer school achievement into middle childhood ^9,11^.

In Palestine, nearly half of children less than two years had IDA^12^. In West Bank (WB), the prevalence of IDA among infants’ aged between 9 to 12 months beneficiaries from maternal and child health centers was 46.6% in 2012 ^13^.

The Palestinian Ministry of Health (PMoH) strategy to prevent and combat IDA includes distributing iron supplements for infants free of charge from six months to two years of age and extended to three years if the child is found anemic^14^. Moreover, a blood test for screening of anemia is requested from all infants at twelve and eighteen months of age while attending the clinic to have immunization.

Despite overall improvements in child health in Palestine, the problem of IDA still exists and hence needs to be studied. So we aimed to estimate the prevalence of IDA and associated risk factors among rural infants.

## Methods

This cross-sectional descriptive study was carried out from 15^th^ of Jan and continued till the 25^th^ of Mar 2015, among infants (aged 9-12 months) and their mothers, at the rural areas of Nablus Governorate, Central Highlands of the West Bank, and north of Jerusalem.

Ethics Committee of High Institute of Public Health in Alexandria University, Egypt, approved the study. Written informed consent was obtained from each participating mother.

The sample size was calculated using EpiInfo version 7.1.2.0. Based on a 46.6% prevalence of anemia among infants in WB (2012)^13^, number of infants aged between 9-12 months in rural areas of Nablus Governorate was 1463 (personal communication), 95% confidence interval, 5% confidence limit, design effect of 2, thirty clusters and a 5% non- response rate, the minimum required sample size was calculated to be 662 infants and their mothers.

Nablus Governorate includes 59 villages. Two villages were excluded because their total number of live births between the beginnings of Dec 2013 till the end of Mar 2014 was zero. Thirty villages were selected randomly from the fifty-seven villages.

The names and birthdates of infants aged between nine and twelve months in each selected village were obtained from the Palestinian Health Information Center. The number of infants within each village was selected randomly and the sample was proportionally allocated according to the total number of infants in each village.

A pre-designed structured interviewing questionnaire was developed to collect socioeconomic and demographic characteristics, pregnancy and birth characteristics and infants feeding habits. The questionnaire was pre-tested on 25 mothers to assess the appropriateness of the format and wording. Based on the results of the pretest, modification in the format of some questions and changing some concepts were done.

With the assistance of trained nurse, venous blood samples were collected from all participating infants and a complete blood count analysis was performed using COULTER® Ac.T diff Hematology Analyzer (BECKMAN COULTER) in order to measure hemoglobin (Hb) concentration. The hematocrit (Hct), mean corpuscular volume (MCV), mean corpuscular hemoglobin (MCH), mean corpuscular hemoglobin concentration (MCHC), and red cell distribution width (RDW) values were also evaluated.

Infants with Hb less than 11 g/dl were considered anemic, and anemia in the study was classified by severity using WHO criteria into the following categories: mild anemia (Hb between 10- 10.9 g/dl), moderate anemia (Hb between 7-9.9 g/dl) and severe anemia (less than 7 g/dl)^15^. The criteria for IDA were Hb less than 11 g/dl and MCV less than 80 fl^16^.

Weight of the infant was measured using Seca digital scale; the body weight was measured to the nearest gram. For length, Seca mobile measuring mat with head and foot braces were used. All infants were without any shoes during the measurement.

Weight and height were analyzed by WHO Anthro 2005 software to calculate z-score of weight for age (WAZ), weight for length (WLZ) and length for age (LAZ). Underweight was defined as WAZ <-2, wasting was defined as WLZ <-2, and stunting was defined as LAZ <-2^17^.

Data obtained from the study were coded and analyzed using the SPSS ver. 20 (Chicago, IL, USA). Results were expressed as frequencies, means and standard deviations (SD) to describe the characteristics of the study sample. Chi-square test (x^2^) used to examine differences with categorical variables. *t*-test was used for comparing the means between groups (with and without IDA). Binary logistic regression analysis was done to test for significant independent predictors for the presence of IDA. Receiver Operating Characteristics (ROC) analysis was performed to evaluate the validity of Hb measurement in screening IDA in infants. *P*<0.05 was considered statistically significant.

## Results

The number of infants enrolled was 660; six infants were excluded because they are thalassemia carriers. In the end, 654 infants with their mothers participated in the study and yielded a response rate of 98.8%.

Table 1 presents the socio-demographic characteristics of the study sample. The mean ±SD age of infants was 11.3±1.34 months. Slightly over half were male (51.1%), only 9.2% of infants were low birth weight. Nearly one quarter was fifth or higher birth order. The mean ±SD mothers’ age was 28.4±5.8 yr with a minimum of 15 yr and a maximum of 45 years. The majority of mothers (92.5%) were housewives. More than half of families (52.8%) were from middle-income households. The prevalence of anemia among mothers during the third trimester was 30.7%, with mean Hb of 9.5±1g/dl among anemic mothers compared to 11.9±0.88 g/dl among non-anemic mothers.

**Table 1:** Socio-demographic characteristics of the study participants (n=654)^a^

**Variables**	**Number**	**Percent**
Infant age (month)		
9-	59	9.0
10-	129	19.7
11-	180	27.5
12-	148	22.6
13-	104	16.0
14	34	5.2
Infant sex		
Male	334	51.1
Female	320	48.9
Birth order		
First	137	20.9
Second	144	22.0
Third	115	17.6
Fourth	89	13.6
Fifth or higher	169	25.9
Mothers age (yr)		
15-24	198	30.3
25-34	352	53.8
≥35	104	15.9
Maternal educational level		
Illiterate	15	2.3
Primary	131	20.0
Secondary	264	40.4
Diploma	49	7.5
Higher education	195	29.8
Family income/month (US$)		
<520	198	30.3
520-1069	345	52.8
>1070	111	16.9
Parity		
1	129	19.7
2	147	22.5
3	115	17.6
4	89	13.6
>5	174	26.6
Spacing (yr)		
First child	137	20.9
<3	270	41.3
>3	247	37.8
Anemia in third trimester		
Yes No	172 389	30.7 69.3
Iron supplement during pregnancy		
Yes	556	85.4
No	95	14.6
Frequency and duration of iron supplements ^b^		
Correct frequency and duration	264	47.5
Incorrect frequency or duration	229	52.5

^a^ Missed data were not included. ^b^ Calculated among mothers who reported receiving iron supplement during pregnancy

The mean Hb concentration of infants was 11.31 ±1.03 g/dl and ranged from 8.1 to 16.1 g/dl. The prevalence of anemia was 37.4% among males and 31.6% among females with no significant difference in terms of sex (x^2^=2.484, *P*=0.115), or in the Hb mean between males and females (*t*=-0.851, *P*=0.390).

The overall prevalence of anemia among infants was 34.6%, where 71.7% of the anemic infants had mild anemia and 28.3% had moderate anemia. No cases of severe anemia were detected. The prevalence of IDA among infants was 32.6% (213 of infants).

**Figure 1:**
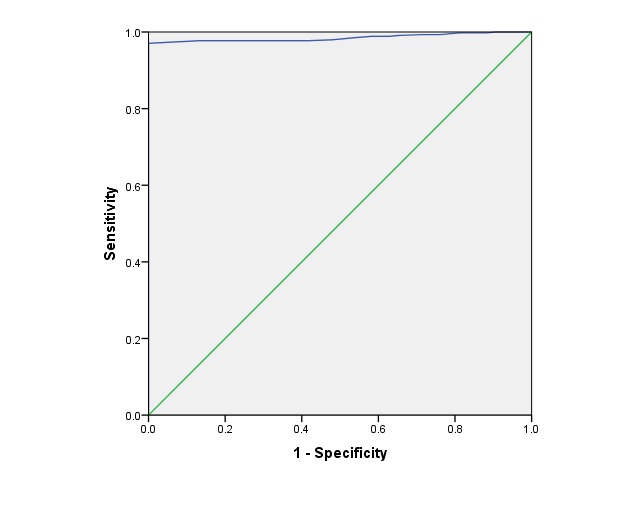
ROC curve analysis for Hb as a predictor of IDA among rural infants

Figure 1 shows the areas under the ROC curve of Hb as a predictor of IDA among rural infants, there is evidence that Hb test has an ability to distinguish between infants with and without IDA (*P*<0.001).

Analysis of dietary history showed that nearly two-thirds of infants (66.4%) were breastfed during the first six months. More than one third (36.7%) had early introduction of complementary food (before 6 months), 45.6% were timely fed (at 6 months). Regarding current type of feeding, less than half (46.6%) of infants have been breastfed. As for infants iron supplements, less than half (45.3%) of infants took iron supplements. Iron deficiency anemia was more prevalent among breastfed infants during the first six months (38.2%), infants who started complementary food after six months (42.2%), infants who still breastfed (41.3%) and among infants who did not take iron supplement with statistical significant difference (Table 2).

**Table 2:** Distribution of rural infants, with and without iron deficiency anemia (IDA), according to their dietary history

**Dietary history**	**IDA**				***P*** **value**
	**Yes (n=213)**		**No (n=441)**		
	**Number**	**Percent**	**Number**	**Percent**	
Type of feeding during the first 6 months					0.001
Breastfeeding	166	38.2	268	61.8	
Partial breastfeeding and formula	41	22.0	145	78.0	
Formula	6	17.6	28	82.4	
Time of introduction of complementary food					0.007
Before 6 months	84	35.0	156	65.0	
At 6 months	80	26.8)	218	73.2	
After 6 months	49	42.2	67	57.8	
Current feeding types					0.001
Did not drink milk	7	36.8	12	63.2	
Breastfeeding	126	41.3	179	58.7	
Formula alone or with breastfeeding	32	18.1	145	81.9	
Fresh cow/sheep milk alone or with breastfeeding	26	36.1	46	63.9	
Powder cow milk alone or with breastfeeding	22	27.2	59	72.8	
Iron supplements					0.038
Yes	84	28.4	212	71.6	
No	129	36.0	229	64.0	

**Figure 2:**
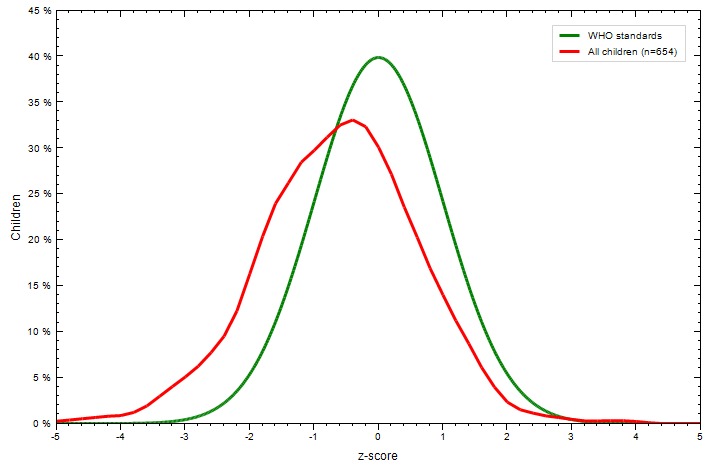
Length for age z-score of rural infants compared to WHO growth standards

The mean ±SD of WAZ, WLZ, and LAZ of the infants were 0.583 ±0.99, 1.16 ±1.06 and -0.614 ±1.21 respectively. Almost all infants had normal WAZ, and WLZ (99.1% and 99.8% respectively), whereas 12.4% of infants were stunted (Figure 2). The mean WAZ was significantly lower (*P*=0.016) among iron-deficient infants (0.449 ±0.97) than among infants without IDA (0.647 ±0.99), both groups had positive WAZ means. The mean WLZ was nearly the same among infants with and without IDA (1.1 ±1.03 and 1.19 ±1.07 respectively), with a statistically insignificant difference. Infants with IDA had significantly (*P*=0.007) lower mean LAZ than infants without IDA (-0.797 ±1.22 and -0.525 ±1.19, respectively), both groups had a negative LAZ.

The mean values of Hb, Hct, MCV, MCH, MCHC and RBCs count revealed significantly higher values among infants without IDA as compared to iron-deficient infants, while the mean value of RDW was significantly higher among iron-deficient infants.

After adjusting for confounders using logistic regression model, significant independent risk factors for IDA were infants being older in age, anemia during the third trimester, birth spacing, breastfeeding during the first six months, early (before six months) and late (after six months) introduction of complementary feeding, and taking iron supplements during pregnancy in an incorrect frequency and/or duration were the significant risk factors for IDA among infants (Table 3).

**Table 3:** Factors associated with IDA among rural infants

**Independent variables**	**Odds ratio** **(95% CI)**	***P *** **value**
Infant age (for every month increased)	1.19 (1.02, 1.40)	0.029
Anemia during third trimester		
No	1.00	
Yes	2.39 (1.55, 3.71)	0.000
Birth spacing (yr)		
First child	1.00	
<3	2.86 (1.58, 5.18)	0.001
≥3	1.81 (0.99, 3.34)	0.055
Exclusive breastfeeding during the first six months	
No	1.00	
Yes	2.40 (1.46, 3.95)	0.001
Introduction of complementary feeding	
At six months	1.00	
Before six months	1.64 (1.03, 2.61)	0.038
After six months	2.26 (1.27, 4.05)	0.006
Frequency and duration of iron supplements during pregnancy
Correct frequency and duration	1.00	
Incorrect frequency and duration	1.18 (1.19, 2.75)	0.006

Sensitivity of the model was 72.1%; CI, confidence interval.

## Discussion

Results of the current study revealed that the prevalence of IDA among rural infants was 32.6%, indicating a problem of moderate public health significance, according to WHO classification^18^. Although the PMoH distributed free iron supplements for infants, the prevalence is still high. This could be attributed to lower mothers’ adherence to iron supplements administration. The most common cause for poor adherence is the taste of iron supplements since the majority of mothers in the study reported when asked about iron supplements that their infants did not accept the taste of the iron supplement distributed by PMoH.

The rate in this study is lower than that reported in Nablus Governorate as 41.8%^19^. This may be attributed to differences between urban and rural areas. On the other hand, the prevalence in Gaza Strip (GS) was much higher. In a study carried out in GS among infants in 2011, the prevalence was 60.4%^20^. The higher rate of anemia in GS compared to WB is due to food security highly dependent on Israeli border closure policies. In 2012, more than one third (1.57 million) of households in Palestine were food insecure after receiving various forms of assistance, with a 7% increase compared to 2011 (from 27% to 34%). WB enjoyed a higher level of food security; 34% versus only 10% in GS. Less than one-fifth of WB households suffered from food insecurity compared to more than half (57%) of the Gazans. The reason behind deterioration in food security was the declined income and increased food prices^21^. Therefore, the traditional Palestinian diet had lost its food diversity and was increasingly centered only on wheat flour, rice, chickpeas, and occasionally some vegetables and foods of animal origin^14^.

The prevalence of IDA in the present study is lower than other studies in Arab countries. In Jordan (2012/2013) the prevalence was 47.4% among toddlers^22^, and it was 51% in Northwestern Saudi Arabia (2015) among children aged 6-24 months^23^.

In developed countries, the prevalence of IDA is much lower (<1%-5.7%)^24-26^. In south-central America and some areas in Asia the figures were higher than that in developed countries but still in the line or lower than that in this study as in Brazil (28.1%, 2009) ^27^, and in China (26.9%, 2010)^28^.

The wide discrepancy between the results in developed and developing countries have been attributed to several factors including differences in the prevalence of anemia-causing illnesses ^29^ and differences in diet; a much lower consumption of food from animal sources observed in developing countries and lower overall nutritional value of the diet compared to developed nations. Fortification of wide range of infants’ food in developed countries played an important role in the reduction of ID^30^.

The present study found that Hb test is a good alternate predictor of IDA in infants and young children. Screening IDA based on Hb measurement is inexpensive and easy, especially in resource-limited setting. This agrees with the WHO and Center for Disease Control and prevention, that the prevalence of anemia may be the best indicator of the iron status of infants and young children^31^.

In addition, increasing age of infants was associated with an increased risk of IDA. The risk of IDA is higher during the second year of life (between 12-18 months) because of increased iron requirements related to rapid growth and improper nutritional practice during the first year of life^32^.

The results of this study support the finding that maternal anemia is a risk factor for IDA among infants during the first year of age ^33,34^. Iron-deficient infants were more likely to have been born with less than three years of spacing (from birth to birth) than infants without IDA. Maternal nutrient depletion due to short intervals between pregnancies will cause a state of biological competition between the mother and the fetus in which the well-being of both is at serious risk ^35^.

According to our study, infants with IDA were more likely to have been breastfed compared to infants without IDA. This result does not contradict with WHO recommendation for exclusive breastfeeding for six months, however, it has its own specificity since they were conducted in settings where maternal anemia are prevalent, and accordingly infants born with insufficient iron stores.

Concerning time of introducing complementary food, we found that early and delayed introduction of complementary food were risk factors for IDA. Early introduction of complementary food does not prevent infants’ IDA. One reason for this is likely to be the poor iron content of complementary food. Since it is impossible to supply enough iron from complementary foods to meet iron needs without high intake of animal products^36^.

Before six months iron required is provided by infants iron stores, and after six months iron requirement must be provided by iron from the diet^37^, delayed introduction of complementary foods can lead to depletion of infants iron stores leading to IDA^38^.

Mothers who did not comply to iron supplement in the correct frequency and duration during pregnancy were more likely to have infants with IDA. This finding is confirmed with the result of a review, that daily dose of iron supplements during pregnancy seems to protect infants from IDA^39^.

One limitation of the study is its cross-sectional design which does not provide a good basis for establishing causality. Moreover, we cannot rule out recall bias since our data regarding infants dietary history is based on mothers recall.

## Conclusion

We evaluated factors directly associated with IDA in infants. Both dietary and non-dietary factors were associated with an increased risk of IDA in infants. Health authorities should interfere to prevent risk factors of IDA by early administration of iron supplements for infants born to anemic mothers, changing the type of iron distributed free of charge at nine months to another type with more acceptable taste. Moreover, the study suggests the need for educational intervention for improving the mothers’ knowledge regarding complementary feeding practices and prevention of IDA in young children.

## Acknowledgements

The authors gratefully acknowledge all municipalities, villages’ councils included in the study, women’s association of Deir Al Hatab Charity, and Beita Development Women’s Association for their cooperation in recruiting the sample. We are also very grateful to all mothers and their infants who participated in this study.

## Conflict of interest statement

All authors declare that they have no competing interests.

## Funding

Not applicable.

## Highlights

- One-third of rural infants in Nablus governorate had Iron deficiency anemia (IDA).
- Birth spacing less than three years is a risk factor for IDA in infancy.
- A significant association was detected between maternal anemia and IDA in infants.
- Hemoglobin test is a good alternative predictor of IDA in infants.
